# AniDriveQA: a VQA dataset for driving scenes with animal presence

**DOI:** 10.3389/frobt.2025.1684845

**Published:** 2025-10-28

**Authors:** Rui Wang, Ruiqi Wang, Hao Hu, Huai Yu

**Affiliations:** 1 The Institute of Computing Technologies, China Academy of Railway Sciences Corporation Ltd., Beijing, China; 2 School of Computer Science and Technology, Xi’an Jiaotong University, Xi’an, Shaanxi, China; 3 The Center of National Railway Intelligent Transportation System Engineering and Technology, Beijing, China; 4 Signal & Communication Research Institute, China Academy of Railway Sciences Corporation Ltd., Beijing, China

**Keywords:** vision-language models, visual question answering (VQA), autonomous driving, animal-involved scenarios, benchmark dataset

## Abstract

**Introduction:**

Animal-involved scenarios pose significant challenges for autonomous driving systems due to their rarity, unpredictability, and safety-critical nature. Despite their importance, existing vision-language datasets for autonomous driving largely overlook these long-tail situations.

**Methods:**

To address this gap, we introduce AniDriveQA, a novel visual question answering (VQA) dataset specifically designed to evaluate vision-language models (VLMs) in driving scenarios involving animals. The dataset is constructed through a scalable pipeline that collects diverse animal-related traffic scenes from internet videos, filters and annotates them using object detection and scene classification models, and generates multi-task VQA labels with a large vision-language model. AniDriveQA includes three key task types: scene description, animal description, and driving suggestion.

**Results:**

For evaluation, a hybrid scheme was employed that combined classification accuracy for structured tasks with LLM-based scoring for open-ended responses. Extensive experiments on various open-source VLMs revealed large performance disparities across models and task types.

**Discussion:**

The experimental results demonstrate that AniDriveQA effectively exposes the limitations of current VLMs in rare yet safety-critical autonomous driving scenarios. The dataset provides a valuable diagnostic benchmark for advancing reasoning, perception, and decision-making capabilities in future vision-language models.

## 1 Introduction

The automotive sector is continuously evolving as manufacturers face pressure to enhance safety, adopt sustainable practices and improve design efficiency (Martínez-Hinojosa et al., 2025). Driven by these challenging requirements, automotive manufacturers are increasingly adopting advanced perception and decision-making systems to support drivers and enable autonomous driving (Toropov et al., 2023). Ensuring the safety and reliability of autonomous driving systems requires robust perception and reasoning capabilities, especially in complex and long-tail scenarios. Among these, driving scenes involving the sudden appearance of animals represent a critical yet underexplored challenge. Animals on or near roadways can cause severe traffic disruptions, accidents, and fatalities. In the United States, approximately 1–2 million collisions between vehicles and large animals occur each year, resulting in significant property damage and human casualties (Donaldson, 2017). The presence of animals poses a serious threat to driving safety. Despite advances in object detection and motion planning, current perception and decision-making systems still struggle to generalize to such rare yet safety-critical events, often due to limited training data and insufficient context-aware reasoning.

Recent advancements in large-scale vision-language models (VLMs) have demonstrated promising generalization abilities across diverse visual scenes. These models leverage extensive knowledge bases and strong reasoning capabilities to interpret complex environments. In the context of autonomous driving, VLMs offer the potential to understand not only what is present in a scene but also to reason about behaviors, predict consequences, and suggest appropriate actions. Their ability to perform zero-shot inference and generate structured outputs makes them particularly suitable for addressing long-tail scenarios involving rare or unexpected entities such as animals.

**FIGURE 1:**
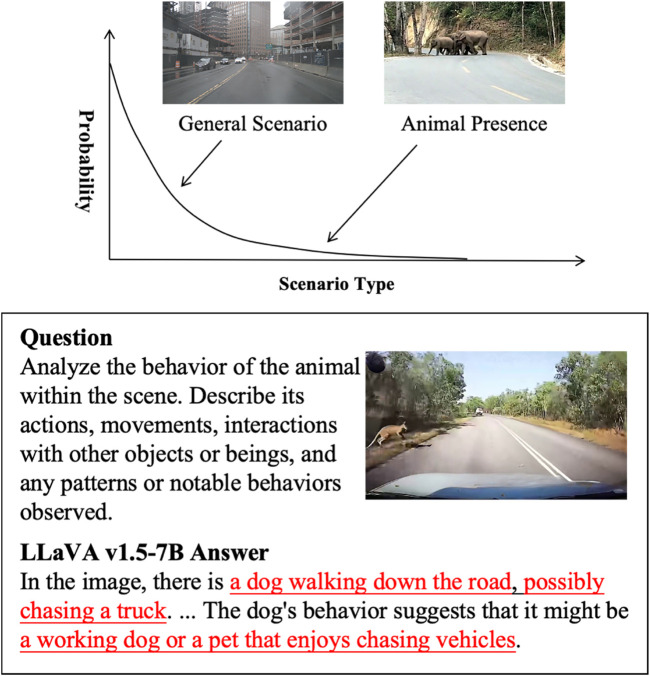
Vision-language model failure in understanding animal-involved driving scenarios.

However, as illustrated in Figure 1, animal-involved driving scenarios represent a typical long-tail phenomenon, where such events occur infrequently in driving data. In these safety-critical cases, existing VLMs may still struggle with scene misinterpretation, incorrect behavior analysis, or unsafe recommendations. Figure 1 provides an example where the LLaVA v1.5-7B model fails to accurately recognize the animal and misjudges the potential driving risk, highlighting the need for dedicated datasets targeting such rare yet impactful scenarios.

Despite the capabilities of VLMs, current datasets for vision-language understanding in autonomous driving primarily focus on general objects, road infrastructure, and human behavior. Datasets such as NuScenes-QA Qian et al. (2024), NuScenes-MQA Inoue et al. (2024), MAPLM-QA Cao et al. (2024), DriveLM-nuScenes Sima et al. (2024), and CODA-LM Chen K. et al. (2025) cover diverse driving scenarios but largely overlook the presence of animals and the reasoning required for safe interaction. For example, CODA-LM contains fewer than 500 images involving animals, making it insufficient for systematic evaluation of animal-related reasoning. This lack of targeted evaluation data limits the ability to assess and enhance model performance in animal-involved driving situations, thereby hindering their deployment in real-world safety-critical conditions.

To bridge this gap, this paper proposed AniDriveQA, a visual question answering (VQA) dataset specifically focused on driving scenarios where animals appear. This paper collected image data from various internet sources and leveraged the capabilities of large vision-language models to generate high-quality question-answer pairs covering key reasoning aspects, including animal detection, behavior recognition, impact analysis, and driving suggestions. The dataset enabled systematic evaluation of VLMs in rare and safety-critical contexts, promoting the development of more robust autonomous driving systems. The main contributions are summarized as follows:

- this paper collected a diverse set of animal-involved driving images and video clips from internet sources, capturing a wide range of species, traffic scenarios, and environmental conditions.
- this paper designed a multi-level visual question answering task suite targeting scene understanding, animal detection, behavior recognition, impact analysis, and driving suggestions.
- this paper constructed the AniDriveQA dataset using large vision-language models for automated question-answer generation.

## 2 Materials and methods

### 2.1 Related works

#### 2.1.1 Autonomous driving datasets

Traditional autonomous driving datasets, such as KITTI Geiger et al. (2013), nuScenes Caesar et al. (2020), Waymo Open Dataset Sun et al. (2020), Cityscapes Cordts et al. (2016), ApolloScape Huang et al. (2018), BDD100K Yu et al. (2020), and Argoverse Chang et al. (2019), primarily focus on visual perception and scene understanding tasks. These datasets provide multimodal sensor data but lack the textual annotations necessary for evaluating higher-level reasoning and decision-making capabilities.

With the emergence of large language models, several datasets have been proposed to introduce linguistic information into autonomous driving. In real-world settings, datasets such as BDD-X Kim et al. (2018), BDD-OIA Xu et al. (2020), Talk2Car Deruyttere et al. (2019), and NuPrompt Wu et al. (2025) extend existing large-scale driving datasets by adding action explanations, natural language commands, and scene descriptions to support reasoning and planning tasks. In simulation environments, CARLA-NAV Jain et al. (2023), Driving-LLM Chen et al. (2024), LaMPilot Ma et al. (2024), and LangAuto Shao et al. (2024) leverage simulators such as CARLA and HighwayEnv to construct datasets combining language instructions with navigation, decision-making, and closed-loop control tasks. Compared to traditional datasets that primarily focus on perception and low-level control, these language-augmented datasets enable a deeper integration of perception, reasoning, and decision-making, offering new opportunities for building explainable and interactive autonomous driving systems. While existing datasets have advanced multi-modal autonomous driving research, they largely overlook rare but safety-critical events involving animals.

#### 2.1.2 VQA tasks in autonomous driving

VQA tasks have become critical components in autonomous driving research, enabling systems to integrate visual perception with natural language reasoning to better interpret complex environments and support decision-making. Existing datasets have designed VQA tasks across multiple dimensions, including perception and scene understanding, sequential reasoning, and high-level decision support. For example, NuScenes-QA Qian et al. (2024) and MAPLM-QA Cao et al. (2024) focus on perception-oriented tasks such as object existence, counting, and spatial relationship recognition. NuScenesMQA Inoue et al. (2024) enhances answer quality by providing responses in fully structured sentences, thereby offering a richer semantic hierarchy. DriveLM Sima et al. (2024) extends VQA to sequential reasoning by modeling the connections between perception, trajectory prediction, and planning. CODA-LM Chen K. et al. (2025) targets decision-oriented reasoning through hierarchical tasks involving scene analysis, regional risk assessment, and driving suggestion generation. Furthermore, datasets like SUTD-TrafficQA Xu et al. (2021), DrivingVQA Corbière et al. (2025), DriveBench Xie et al. (2025), Rank2Tell Sachdeva et al. (2024) and LingoQA Marcu et al. (2024) explore higher-order reasoning tasks such as event forecasting, causal explanation, counterfactual inference, and driver attention modeling. NuScenes-SpatialQA Tian et al. (2025) is designed for both spatial understanding and spatial reasoning in autonomous driving. In addition, STRIDE-QA Ishihara et al. (2025) defines object-centric spatial, ego-centric spatial, and ego-centric spatiotemporal QA tasks to support fine-grained, predictive reasoning in complex traffic scenarios. AutoTrust Xing et al. (2024) focuses on the influence of trustworthiness factors, such as safety, privacy, and robustness, on the operational performance and reliability of autonomous driving systems across diverse driving scenarios. While these efforts significantly advance multimodal reasoning in autonomous driving, rare yet safety-critical situations–particularly those involving animals–remain largely underexplored.

### 2.2 Methodology

Animal appearances in driving environments are highly unpredictable, making it costly and inefficient to capture such data through real-world collection. Inspired by the pretraining strategies of large language models, this paper explored internet video platforms as a rich source for animal-involved driving scenes, offering diverse species, lighting conditions, and geographic contexts. This research was conducted at Institute of Computing Technologies, China Academy of Railway Sciences Corporation Ltd., Beijing, China.

This paper manually collected videos from Bilibili and YouTube via keyword search and verified them before batch downloading with yt-dlpyt-dlp Contributors, (2021). Frames were extracted at fixed intervals to build an initial pool. A multi-stage filtering pipeline was then applied: YOLOv5x Jocher (2020) and Grounding DINO Liu S. et al. (2024) detected animals, Places365 Zhou et al. (2017) classified road-related scenes, and CLIP ViT-B/32 Radford et al. (2021) evaluated semantic relevance. Frames passing these stages were manually reviewed for quality assurance.

Based on the alignment between images and textual descriptions, we further filter part of the data to verify whether the image content matches the target semantics. After manual verification, we obtain approximately 12K images that meet the requirements. In addition, during the above data mining process, the results of object detection and scene recognition on the images are stored as pre-annotations, providing references for the subsequent construction of visual question-answering data. The specific video sources and image quantities are summarized in Table 1, while the distributions of animal categories and scenes are shown in Figures 5a,b, respectively.

**TABLE 1:** Statistics of data sources.

Video platform	Number of videos	Total images	Valid images
Bilibili	205	58,323	10,372
YouTube	31	18,796	2,448

**FIGURE 2:**
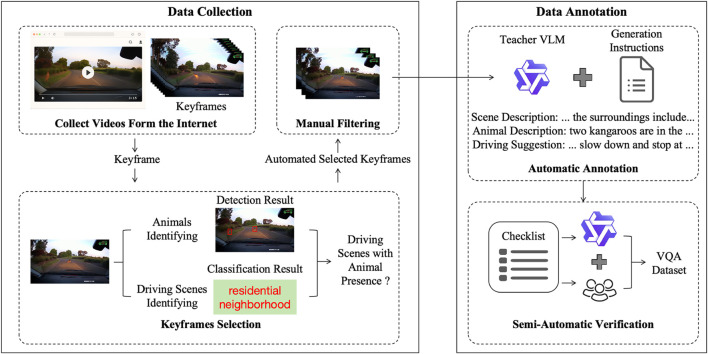
Data construction pipeline.

For annotation, preliminary object and scene information were used as pseudo-labels to assist downstream VQA construction. This paper established a large language model-driven semi-automated validation process with human involvement. The overall data construction process is illustrated in Figure 2.

#### 2.2.1 VQA tasks & metrics

**FIGURE 3:**
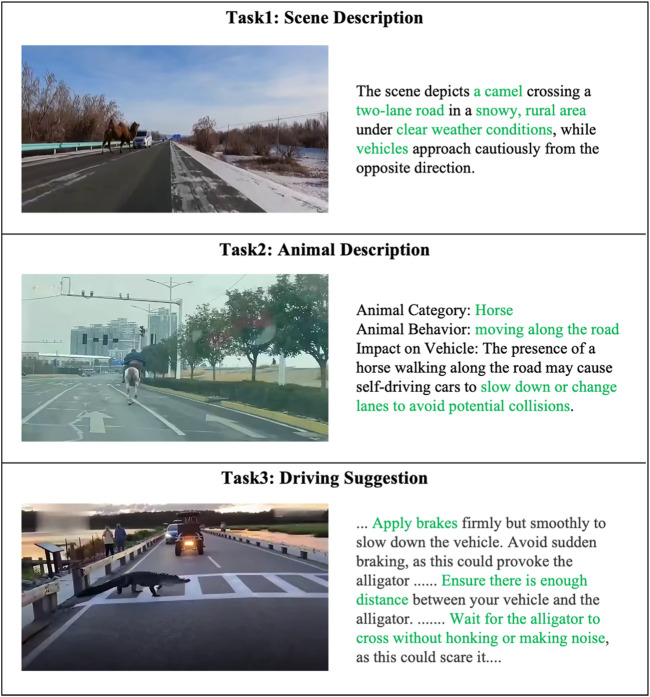
Overview of VQA tasks in AniDriveQA.

The VQA tasks in AniDriveQA are designed to evaluate vision-language models’ capabilities in scene understanding, animal recognition, and decision-making under complex driving conditions. An overview of the tasks is illustrated in Figure 3.

##### 2.2.1.1 Scene description task

The scene description task requires models to generate a comprehensive summary of the driving environment, covering elements such as road conditions, weather, scene type, and key participants like vehicles and pedestrians. As illustrated in Task 1 of Figure 3, this task is not limited to identifying common traffic participants but also requires providing detailed descriptions of abnormal or infrequent factors that may pose significant safety risks.

##### 2.2.1.2 Animal description task

The animal description task focuses on identifying the species, describing the behavior (e.g., stationary, crossing the road), and assessing the potential influence of the animal on the ego vehicle’s driving decisions, reflecting the model’s ability to extract fine-grained, behavior-aware information. As illustrated in Task 2 of Figure 3, the presence of a horse moving along the road requires the model to not only detect the animal itself but also reason about the possible consequences for driving safety, such as prompting self-driving cars to slow down or change lanes to avoid potential collisions, highlighting the integration of perception and reasoning in various traffic scenarios.

##### 2.2.1.3 Driving suggestion task

The driving suggestion task evaluates the model’s reasoning ability by requiring it to propose concrete and safety-oriented driving recommendations based on the scene context, particularly considering the animal’s presence and behavior. This task therefore emphasizes the model’s ability to integrate perception with decision-making and to provide actionable guidance in various scenarios. As shown in Task 3 of Figure 3, when an alligator crosses the road, the model should suggest safe actions such as braking smoothly, keeping distance, and waiting quietly until the animal passes.

##### 2.2.1.4 Evaluation metrics

The evaluation of models on AniDriveQA covers both closed-form and open-form VQA tasks, classified based on the nature of their expected responses. Closed-form tasks, including animal species recognition and behavior classification, have a finite set of possible answers and are evaluated using classification accuracy, as defined in Equation 1:

$$\text{Accuracy}=\frac{N_{\text{correct}}}{N_{\text{total}}}$$ (1)

where $N_{\text{correct}}$ represents the number of samples the model predicted correctly, and $N_{\text{total}}$ represents the total number of samples.

For open-form tasks, including scene description, animal impact analysis, and driving suggestion, model responses are free-form and diverse. Since traditional lexical overlap metrics such as BLEU Papineni et al. (2002) and CIDEr Vedantam et al. (2015) are insufficient to fully capture the semantic quality and reasoning depth of these responses, this paper adopted a large language model (LLM)-based evaluation approach, inspired by recent works (Zheng et al., 2023; Lin and Chen, 2023. Specifically, this paper utilized DeepSeek R1 14B Guo et al. (2025) to conduct prompt-guided evaluations, scoring each response on a 1–10 scale against reference answers and task-specific criteria. Scene description is evaluated based on the accuracy, completeness, and clarity of environmental depiction. Animal impact analysis focuses on the clarity, relevance, and logical soundness of the inferred impact on driving behavior. Driving suggestion assessment considers the reasonableness, safety, contextual adaptability, and clarity of the recommended actions. This hybrid evaluation strategy ensures a comprehensive assessment of both recognition capabilities and complex reasoning abilities critical for safe autonomous driving.

#### 2.2.2 Annotation process

To support the use of AniDriveQA for both training and evaluation, each image depicting an animal-involved driving scenario must be annotated with VQA data aligned to the designed tasks. High-quality annotations are essential for ensuring the accuracy and consistency of model assessment. However, manual annotation is labor-intensive and impractical for large-scale datasets. To address this, this paper developed a multi-step, task-decoupled annotation pipeline, enhanced by a large language model-driven semi-automated validation process, which significantly improves annotation quality and efficiency.

**FIGURE 4:**
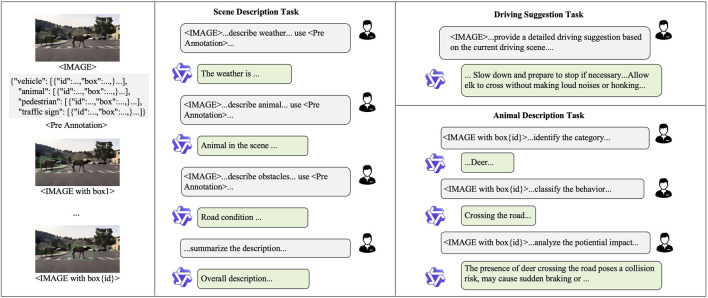
Data annotation pipeline.

As illustrated in Figure 4, the annotation process begins with object detection to identify entities such as vehicles, pedestrians, animals, and traffic signs. The detection results are stored in structured JSON format and serve as context for guiding the vision-language model Qwen-VL 72B Bai et al. (2023) in generating task-specific answers. Each VQA task is handled independently with tailored prompts: for the scene description task, the model synthesizes a textual summary covering weather, road conditions, and key participants; for the animal description task, it extracts each detected animal, identifies its species, determines its behavior, and analyzes its potential impact on driving decisions; for the driving suggestion task, it reasons over the scene and entities to generate a concise, context-aware recommendation.

All answers are structured into a unified format and undergo a semi-automated validation phase. A large model first inspects the annotations based on a predefined checklist, identifying potential errors and suggesting corrections, which are then reviewed and refined by humans. This hybrid validation strategy balances efficiency with annotation quality, ensuring that the final outputs serve as reliable supervision signals for model training and evaluation.

#### 2.2.3 Statistics

The AniDriveQA dataset contains a total of 12,820 samples. To facilitate both evaluation and finetuning, this paper splits the AniDriveQA dataset evenly into training and testing sets with a 1:1 ratio. The testing set serves as the benchmark for model evaluation, while the training set is used for adaptation experiments.

**FIGURE 5:**
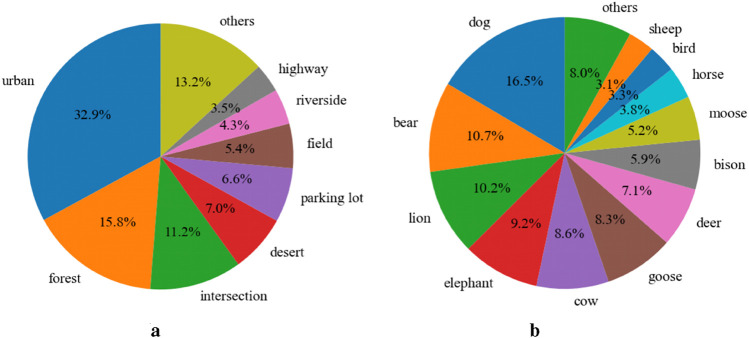
Distribution of scenarios and animal types in the dataset. **(a)** Scenario statistics. **(b)** Animal statistics.

To demonstrate the diversity of AniDriveQA, this paper provided a statistical overview of the animal species and driving scene types covered in the dataset. As shown in Figures 5a,b, the dataset includes a broad range of animal categories, from commonly seen species such as dogs and deer to rarer or region-specific animals like moose and elephants. This ensures the inclusion of both frequent and long-tail classes that pose unique challenges to autonomous driving systems.

The driving scenes in AniDriveQA span various environmental contexts, including urban roads, forests, intersections, and off-road locations such as deserts and riversides. This scene-level diversity reflects the real-world complexity in which animals may appear and allows for a comprehensive evaluation of vision-language models across a spectrum of traffic conditions and geographies.

## 3 Results

### 3.1 Implementation details

The methodology employed in this study involves several key components. Data annotation was performed using multi-step reasoning workflows with large language models. Video data were collected via web crawling using yt-dlp, and the Qwen-72B model was deployed locally to perform multi-step reasoning for annotation. For model fine-tuning, LLaVA-v1.5-7B was used as the base model, with LoRA fine-tuning (rank = 8). The LLMFactory toolkit was utilized for large model training. All experiments were conducted on an NVIDIA RTX A6000 GPU with 48 GB memory.

To validate the effectiveness of DeepSeek R1 14B scoring, we had two independent raters manually score the animal impact and driving scene labels for samples generated by two VLM models, LLaVA-1.5-13B (175 samples) Liu et al. (2023) and DeepSeek-VL2-Small (151 samples), on a scale of 1–10. We then computed the Cosine Similarity and Pearson Correlation between these scores and those produced by DeepSeek R1 Guo et al. (2025), to validate the effectiveness of DeepSeek R1’s scoring and its agreement with human ratings. Additionally, we calculated the Cosine Similarity and Pearson Correlation between the scores from the two raters.

LMSYS Zheng et al. (2024a) pointed out the feasibility of using GPT-4 as an evaluator to score question-answering results on a 1–10 scale, showing a high consistency with human evaluations. In addition, LLM-EVAL Lin and Chen (2023) proposed a multidimensional evaluation method for open-domain QA, achieving more comprehensive assessments through prompt engineering. Consistent with these findings, it can be seen in Tables 2, 3 that although DeepSeek’s scores have a lower Pearson correlation with human ratings than the correlation between the human raters themselves, they still show a strong positive correlation, and the cosine similarity is extremely high.

**TABLE 2:** Cosine similarity and Pearson correlation for each ScoreType-Model-Human combination.

ScoreType	Model	Human	Cosine similarity	Pearson correlation	Sample number
Animal impact	DeepSeek-VL2-Small	Person 0	0.978	0.231	151
Animal impact	DeepSeek-VL2-Small	Person 1	0.953	0.133	151
Animal impact	LLaVA-1.5-13B-HF	Person 0	0.978	0.178	175
Animal impact	LLaVA-1.5-13B-HF	Person 1	0.982	0.234	175
Driving scene	DeepSeek-Vl2-Small	Person 0	0.976	0.358	151
Driving scene	DeepSeek-VL2-Small	Person 1	0.932	0.432	151
Driving scene	LLaVA-1.5-13B-HF	Person 0	0.981	0.322	175
Driving scene	LLaVA-1.5-13B-HF	Person 1	0.979	0.277	175

**TABLE 3:** Aggregated similarity and correlation between two human annotators for each ScoreType.

ScoreType	Model	Cosine similarity	Pearson correlation	Sample number
Animal impact	LLaVA-1.5-13B-HF	0.986	0.585	175
Animal impact	DeepSeek-VL2-Small	0.971	0.388	151
Driving scene	LLaVA-1.5-13B-HF	0.985	0.457	175
Driving scene	DeepSeek-VL2-Small	0.954	0.595	151

### 3.2 Main results

This paper evaluated eight open-source vision-language models on AniDriveQA, including five 7B-scale models (e.g., LLaVA-1.5-7B, Qwen2.5-VL-7B) and three 13B-scale models (e.g., LLaVA-1.5-13B, InstructBLIP-Vicuna-13B). All models are tested under a zero-shot setting with the same prompt template and evaluated following the metrics described above. To facilitate comparison, subjective scores originally rated on a 1–10 scale are linearly scaled to a 1–100 range. The evaluation results are summarized in Table 4.

**TABLE 4:** Performance of open-source VLMs on the AniDriveQA dataset. Bold and underlined values denote the highest and second-highest scores per column.

Model	Scene description text score	Species recognition accuracy	Behavior recognition accuracy	Impact analysis text score	Driving suggestion text score
Qwen2.5-VL-7B Bai et al. (2025)	70.42	0.64	0.77	63.88	64.39
MiniCPM-Llama3-V2.5 Yao et al. (2024)	59.03	0.59	0.67	66.83	66.10
LLaVA-1.5-7B Liu et al. (2023)	51.77	0.47	0.12	50.25	52.16
InternLM-XComposer2.5-7B Zhang et al. (2024)	60.01	0.59	0.87	**71.77**	**69.59**
Janus-Pro-7B Chen et al. (2025b)	54.33	0.55	0.24	54.92	58.32
LLaVA-1.5-13B Liu et al. (2023)	54.67	0.48	0.18	52.90	56.49
InstructBLIP-Vicuna-13B Liu et al. (2024a)	46.05	0.51	0.08	49.80	51.24
Ovis-Clip-Qwen1.5-14B Lu et al. (2024)	65.19	0.57	0.65	58.92	61.98
LLaVA-1.5-7B (Fintuned)	**72.87**	**0.74**	**0.87**	70.96	68.17

The results reveal notable differences among models. Qwen2.5-VL-7B achieves the best overall performance, demonstrating strong abilities in both perception and reasoning tasks, with leading results across scene description, species recognition, and driving suggestion. It consistently outperforms other models in both closed-form classification and open-form generation tasks. Other models, such as Ovis-Clip-Qwen1.5-14B and MiniCPM-Llama3-V2.5, perform competitively on closed-form tasks, each achieving species recognition accuracies around 0.6 and maintaining reasonable behavior recognition performance. In contrast, models like LLaVA-1.5 and InstructBLIP-Vicuna-13B show significant limitations in fine-grained reasoning, with behavior recognition accuracies dropping below 0.2, and lower text quality scores in impact analysis and driving suggestion tasks.

**FIGURE 6:**
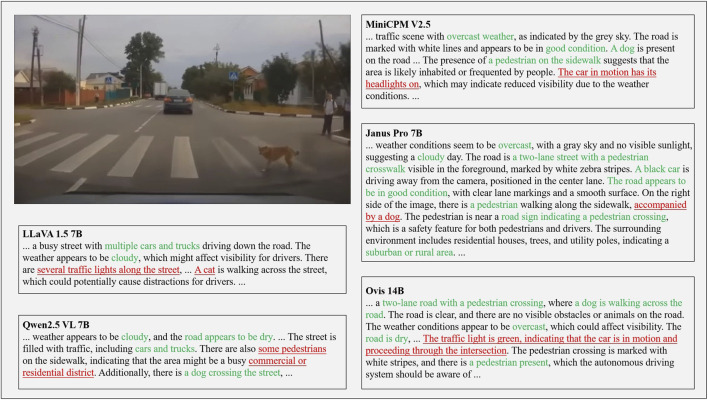
Example outputs generated by different vision-language models.

These results highlight that while some models perform reasonably on basic recognition tasks, they struggle when deeper semantic understanding and contextual reasoning are required. The results also demonstrate that AniDriveQA effectively distinguishes models’ abilities in perception and reasoning specifically under animal-involved driving scenarios, providing a targeted benchmark for evaluating vision-language models in safety-critical and rare-event contexts. Representative qualitative outputs from selected models are illustrated in Figure 6.

To further investigate data adaptability, this paper finetuned LLaVA-1.5-7B model on AniDriveQA using the llamafactory toolkit Zheng Y. et al. (2024) and the LoRA technique (Hu et al., 2022). During training, the multimodal projection layers are frozen to maintain visual feature extraction capabilities, while the core LLM and visual encoder parameters are updated. The training process adopts a context window of 4096 tokens, employs a linear warm-up followed by cosine decay across five epochs, and leverages mixed-precision training to improve efficiency.

In addition to finetuning on AniDriveQA alone, this paper explored a mixed-data strategy by combining AniDriveQA with the CODA-LM benchmark. This paper designed three experimental settings: finetuning solely on AniDriveQA, finetuning solely on CODA-LM, and finetuning on the combined dataset. For evaluation, AniDriveQA tasks are assessed using the same methodology described earlier, combining classification accuracy for closed-form tasks and LLM-based scoring for open-form tasks, while CODA-LM tasks are evaluated using its original evaluation protocol. The results are summarized in Table 5.

**TABLE 5:** Comparison of finetuned models on the AniDriveQA dataset and CODA-LM benchmark. Bold and underlined values denote the highest and second-highest scores per column.

Model	AniDriveQA					CODA-LM		
	Scene description text score	Species recognition accuracy	Behavior recognition accuracy	Impact analysis text score	Driving suggestion text score	General description text score	Regional awareness text score	Driving suggestion text score
LLaVA-1.5-7B	51.88	0.47	0.12	59.36	61.14	19.30	42.06	23.16
Finetuned on AniDriveQA	72.87	0.74	0.87	**70.96**	68.17	30.54	38.28	49.15
Finetuned on CODA-LM	70.34	0.42	0.77	61.24	64.40	54.87	**74.90**	57.38
Finetuned on Mixed Data	**74.80**	**0.75**	**0.87**	68.08	**71.17**	**59.04**	64.50	**60.36**

Finetuning solely on AniDriveQA significantly enhances model performance in animal-involved scenarios, highlighting the importance of targeted rare-event data. Finetuning on CODA-LM improves general driving scene understanding but shows limited gains in handling rare animal-related events. Finetuning on the combined dataset achieves the best overall results, suggesting that integrating both traditional and rare driving scenarios enables models to better generalize across common and safety-critical conditions.

Overall, these experiments demonstrate that AniDriveQA is not only a challenging benchmark for evaluating vision-language models in complex autonomous driving scenarios but also an effective resource for improving model robustness and reasoning through finetuning. By rigorously testing models across both closed-form and open-form tasks, AniDriveQA promotes the development of safer and more intelligent autonomous driving systems capable of handling unpredictable real-world situations.

## 4 Discussion

To better compare our dataset, we evaluate it against the latest similar datasets, as shown in Table 6. Our dataset significantly differs from prior works such as CODA-LM Chen K. et al. (2025) and SUP-AD Sima et al. (2024). Specifically, while CODA-LM Chen K. et al. (2025) contains fewer than 500 scenes with animals and lacks detailed animal information, and SUP-AD Sima et al. (2024) does not provide driving advice or animal information, our dataset contains 12,000 scenes with animals and provides comprehensive annotations including scene descriptions, object descriptions, driving advice, and animal information. This richer annotation enables more detailed analysis and model evaluation, addressing limitations in prior datasets. By incorporating these additional dimensions, our work facilitates more thorough comparisons and insights, thereby enhancing the scope and utility of the results.

**TABLE 6:** Comparison of datasets.

Dataset	Scenes with animals	Scene desc.	Object desc.	Driving sug.	Animal info.
CODA-LM Chen et al. (2025a)	< 500	✓	✓	✓	×
SUP-AD Sima et al. (2024)	-	✓	✓	×	×
Ours	12K	✓	✓	✓	✓

This paper proposed AniDriveQA, a novel visual question answering dataset specifically designed to evaluate the reasoning capabilities of vision-language models in animal-involved driving scenarios. The dataset is constructed through a semi-automated pipeline that combines internet video mining with LLM-based annotation. It covers a diverse set of tasks, including scene description, animal description, and driving suggestion. Comprehensive experiments on a range of open-source models demonstrate the effectiveness of AniDriveQA in revealing the strengths and limitations of current vision-language models in complex and safety-critical scenarios. Nevertheless, deployment remains challenging, and future work should improve model robustness and efficiency to ensure safe and reliable performance under rare animal appearances, real-time constraints, and diverse driving conditions.

This work fills a critical gap in autonomous driving research by introducing a benchmark dataset that systematically evaluates vision-language models in rare but safety-critical animal-involved scenarios, advancing the study of long-tail perception and reasoning. Animal-related traffic accidents cause substantial human, economic, and ecological losses each year, and this research contributes to safer and more reliable autonomous driving systems by enabling more robust perception and decision-making in such scenarios.

## Data Availability

The dataset AniDriveQA for this study can be found at https://pan.baidu.com/s/1MyUQkr3OuKIJyyTQ2HI_EQ, with the extraction code r2in.
